# Multicentric Glioma: An Ideal Model to Reveal the Mechanism of Glioma

**DOI:** 10.3389/fonc.2022.798018

**Published:** 2022-06-07

**Authors:** Yong Yan, Wei Dai, Qiyong Mei

**Affiliations:** Departmentof Neurosurgery, Changzheng Hospital, Naval Medical University, Shanghai, China

**Keywords:** multicentric glioma, ideal model, stem-cell-origin theory, tumorigenicity, stemness

## Abstract

As a special type of glioma, multicentric glioma provides an ideal pathological model for glioma research. According to the stem-cell-origin theory, multiple lesions of multicentric glioma share the same neuro-oncological origin, both in gene level and in cell level. Although the number of studies focusing on genetic evolution in gliomas with the model of multicentric gliomas were limited, some mutations, including IDH1 mutations, TERTp mutations and PTEN deletions, are found to be at an early stage in the process of genetic aberrance during glioma evolution based on the results of these studies. This article reviews the clinical reports and genetic studies of multicentric glioma, and intends to explain the various clinical phenomena of multicentric glioma from the perspective of genetic aberrance accumulation and tumor cell evolution. The malignant degree of a glioma is determined by both the tumorigenicity of early mutant genes, and the stemness of early suffered cells.

## Introduction

Gliomas are the most common primary malignant tumors in the brain, of which glioblastoma (GB) accounts for 60-70% and is the most malignant type of gliomas, with a median survival time of only 12-15 months (1). It was once believed that a glioma is a homogeneous entity, with tumor cells centered on the tumor nest and distributed in a scattered and radiating manner. However, the tumor stem cell theory overturns this theory of tumor homogeneity and proposes that the tumor population is more like a family (2, 3). Inside the tumor, there is also the differentiation of stem cells into precursor cells at various levels, but due to genetic defects, they cannot differentiate into normal mature neural or glial cells, which lead to the formation of tumors (4, 5). Glioblastomas mostly recur in situ, which can be explained by the recurrence of tumor cells that have not been completely removed by surgery and other treatments at the edge of the tumor. However, in a small number of patients, ectopic recurrences in one or multiple sites also exist. It is true that some ectopic recurrences can be explained by tumor migration along the white matter tract or by the spread of cerebrospinal fluid, but there are indeed some ectopic recurrences that cannot be explained by known metastasis pathways. This phenomenon is actually a form of multicentric glioma (MCG), i.e., metachronous MCG.

Multicentric gliomas are gliomas that occur simultaneously or successively in multiple parts of the central nervous system, and the lesions are not connected and cannot be explained by the existing ways of tumor metastasis like white matter tract migration or cerebrospinal fluid spreading (6). In order to explain the pathogenesis of MCG, there are many theories including *de novo* occurrence (7), metastasis (8), and stem cell origin (9). The *de-novo*-occurrence theory believes that the lesions occur separately, and there is no correlation in genetic background among them. The population incidence rate of glioma is 6.6 per 100,000, so the probability of two gliomas with completely unrelated genetic backgrounds in the same patient is 4.4/10^9^, and the probability of more than three occurrences at the same time is almost zero. However, in fact, MCG reported in the literature account for about 1-10% of central nervous system gliomas (10–17), which is 6.6-66/10^7^. The number is much higher than the calculated data with the claim of the theory of *de novo* occurrence. The metastasis theory believes that other lesions are transferred from an early lesion in some way. However, the genomic analysis of multiple foci of MCG found that there are large differences of genetic mutations among the foci, which cannot be explained by the metastasis theory. The stem cell origin theory may be a more reasonable explanation for the mechanism of MCG. Neural stem and progenitor cells are distributed in neural stem cell pools such as the subventricular zone (SVZ) and subgranular zone (SGZ) (18). In normal conditions, these cells differentiate into various neural progenitor cells, and finally into mature neurons or glial cells (19). The stem-cell-origin theory believes that one or more critical gene mutations may have occurred in some stage of neural precursor cells. In the subsequent migration and differentiation process, these genetically defective precursor cells gradually developed into various gliomas (4, 5). It is speculated that the migration and differentiation are in a single direction in most cases, so most gliomas are solitary; but occasionally, the process is in multiple directions, thus leading to the appearance of MCG. For many years, people have been looking for key genes or molecular targets for tumorigenesis from the perspective of gene mutation and abnormal expression. Although high-throughput genetic screening, survival analysis and bioinformatics techniques can be used to find some early mutations, such as IDH1/2 (20–23), a considerable proportion of gliomas have not been found to have a clear source mutation. Multicentric glioma is a special pathological phenomenon. If the hypothesis that different lesions of MCG share the same source mutation is established, then the greater the difference in genetic background among the lesions, the less the shared mutation genes will be. Therefore, it is more likely to find the source mutation in these shared mutation genes. For this reason, MCG provides us with an excellent model, which realizes the early screening of shared mutations between lesions through natural mechanisms, which may provide a shortcut for discovering mutation genes in a very early stage of glioma-genesis. As an ideal pathological model, multicentric glioma deserves further understanding and attention.

## Definition, Classification and Epidemics of MCG

Gliomas with multiple lesions in the brain need to differentiate between multifocal glioma (MFG) and MCG. In the former, multiple lesions can be explained by known metastasis pathways, such as white matter fiber tracts, cerebrospinal fluid pathway, or local metastasis; in the latter, the relationship between multiple lesions cannot be explained by the above ways (6, 8). Metastatic lesions have a genetic background of high consistency, while the genetics of multicentric lesions have more differences.

The definition of MCG has been developing. The disjointness of the lesions on the MRI FLAIR was used to exclude MFG (10, 24, 25). In the future, the diagnosis of MCG or MFG may mainly rely on molecular genetic studies. By analyzing the molecular genetic background of multiple lesions in a certain case, an individualized molecular genetic evolutionary tree can be drawn, so that all lesions can be marked at different positions in the evolutionary tree. The definition of MCG and MFG mainly relies on the position on the molecular genetic evolutionary tree, rather than the imaging or anatomical connection. When the results of molecular genetics and imaging anatomy appear to be contradictory, the former should be followed instead of the latter [as case reported by Reis et al. (7) and Akimoto et al. (26)]. The boundary of MCG and MFG may become blurred, and between the two extremes there will be some gray areas that are difficult to clearly classify.

## Incidence

The incidence of MCG is reported between 1-10%. Salvati et al. summarized 14 years of glioma cases, and found that MCG accounted for about 2% of gliomas diagnosed and treated in the same period (10). There are many reasons why it is difficult to determine its accurate incidence. One is that genetic testing is not yet popularly used to distinguish MCG from MFG; the second is that some MCG are metachronous, which may be missed due to loss to follow-up; the third is the neglect of small ectopic lesions. Lasocki et al. analyzed the magnetic resonance images of patients with GB and found that 6% of the cases had small multicentric non-enhancing brain lesions, and a part of them progressed to new lesions subsequently (27).

## Time and Space

Multiple lesions may exist simultaneously at the time of diagnosis (synchronous MCG), or they may appear one after another in a period of time (metachronous MCG). The longest interval between the appearance of lesions reported is 22 years (28).

**Figure 1:**
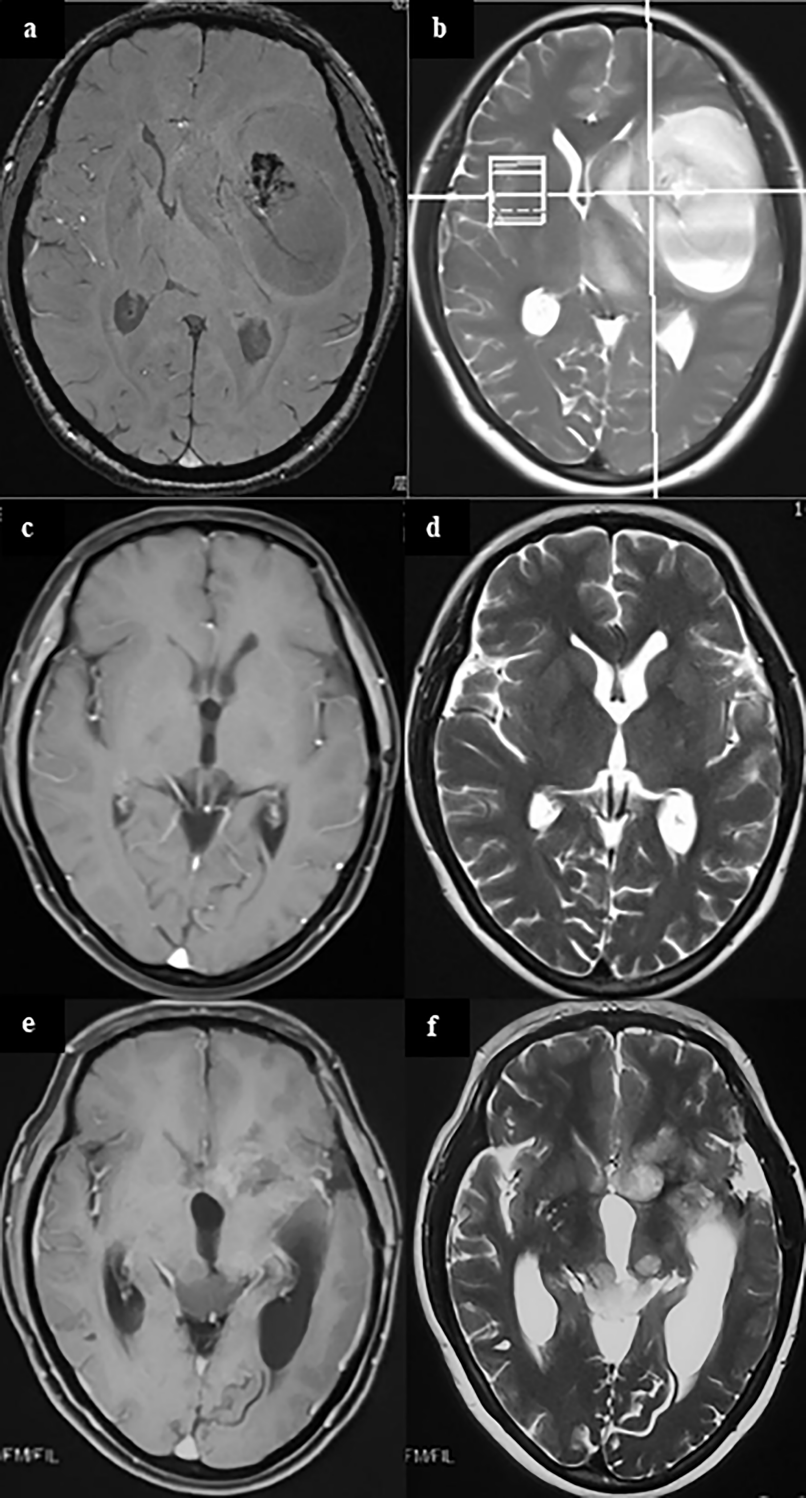
A case of metachronous multicentric glioma. The patient underwent MRI for headache and found a tumor in the left temporal insula **(A, B)**. She was given partial resection of the tumor. Pathology showed GBM. Local radiation and temozolomide based chemotherapy were given after surgery. The tumor completely regressed in MRI 1 year after surgery **(C, D)**. However, in follow-up 1.5 years after surgery, it was found that the patient had a recurrent lesion in the left caudate head and insula, and an ectopic lesion in the midbrain tectum **(E, F)**. On sequential T2 and FLAIR MR images, there was no connection between the lesion in the midbrain tectum and in the right basal ganglia. In addition, cytologic test of CSF and MR of the brain and spinal cord did not find there was any evidence of CSF dissemination. Therefore, the lesion in the midbrain tectum is considered as a metachronous multicentric glioma in contrast with the initial tumor. The patient underwent ventricular-abdominal shunt surgery to relieve hydrocephalus and was given temozolomide chemotherapy again. She is still under follow-up (21 months after tumor resection). (a,c,e, MRI of T1 enhanced signal; b, d, f, MRI of T2 signal).

Interestingly, metachronous MCG has a longer survival time than ordinary solitary glioma [median survival time 353 days vs. 234 days, p<0.05 (29)]; but once ectopic recurrence occurs, the survival time is similar to that of synchronous multicentric glioma, with a median survival time of only 7 months (25). In our clinical experiences, we also find patients with ectopic recurrences usually have a longer survival time. In some cases, even when the tumor progresses, there is no tumor recurrence at the original site, but a tumor growing at a new site (**Figure 1**). We speculate that this may be because the location of the primary lesion is easier to obtain more thorough surgical resection, or it is more sensitive to radiation or chemo-therapies.

At the age of onset, although most MCG occur in adults aged 30-70, they can also occasionally occur in children (8, 30–32).

Similar to solitary gliomas, MCG can occur in various parts of the nervous system, including supratentorial (11), subtentorial and introspinal (33–35). The most common site is supratentorial. It can involve left, right or both cerebral hemispheres. The probability of multiple lesions on one side is about twice that of bilateral (10).

## Pathological Types

The common pathological types of MCG include glioblastoma multiforme (GBM), anaplastic astrocytoma(AA), and low-grade astrocytoma, of which GB is the most popular. The 2021 WHO Classification of CNS Tumors has cancelled the terminology of primary/secondary GBM and AA, and substitutes them with Glioblastoma, IDH-widetype, or Astrocytoma, IDH-mutant, grade 3 and 4. However, due to most of the cases of MCG are reported before the publication of the new classification, the terminologies of GBM and AA are continued to use here. Most of the lesions have the same or similar pathological grade, such as GB, GB combined with AA, or all low-grade astrocytoma, but there are also a few cases diagnosed as high-grade glioma combined with low-grade glioma. In these cases, the median progression-free survival (PFS) time is 9 months (36), which is similar to high-grade MCG. Special types include epithelioid GB (37), optic glioma (37–39), ganglion glioma (40, 41), pilocytic astrocytoma (28, 29), and oligodendroglioma (28) (clinical and pathological features are summarized in **Table 1**).

**Table 1:** Clinical features and genetic aberrance of MCG reported in literature.

Authors	Publish year	Number of cases	Gender	Age (median and range)/yr	S/M MCG	Pathology	WHO grade	Surgery	Radiotherapy	Chemotherapy	OS(median and range)/months	Genetic aberrance	Source mutations
Solitare et al. (42)	1962	1	f	15	na	DA	2	r	n	n	60	na	na
Batzdorf et al. (6)	1963	2	m2	44/62	na	GBM1/AA1	4/3	r2	y2	y2	24/30	na	na
Solomon et al. (43)	1969	1	f	32	S	OA&DA	2&2	r	y	n	36	na	na
Chadduck et al. (44)	1983	1	m	63	S	GBM	4	b	y	n	5	na	na
Kato et al. (45)	1990	1	f	59	S	AA	3	r	y	n	1	na	na
Pell et al. (46)	1991	1	m	11	S	GBM	4	b	y	n	1	na	na
Philippon et al. (47)	1992	1	f	53	na	PA&GBM	1&4	y	n	n	8	na	na
Mamelak et al. (48)	1994	5	m4/f1	11 (3–43)	M5	PA4,O&PA1	1,2&1	r3/b2	y5	y3/n2	all alive 30 (21–37)	na	na
Sim et al. (49)	1999	1	f	11	S	O&PA	2&1	r	n	n	alive at 30	na	na
van Nielen et al. (50)	1999	1	m	28	S	DA	2	b	y	n	Alive at 6	na	na
Franco et al. (51)	2000	2	f2	39/52	M/S	GBM/DA	4/2	r/b	y/n	y/n	20/16	na	na
Reis et al. (7)	2001	1	m	54	M	AA&GBM	3&4	r	y	y	10yr	TP53, PTEN, EGFR, p16 deletion	na
Zamponi et al. (8)	2001	1	m	12	S	AA	3	r	y	n	na	na	na
Synowitz et al. (38)	2002	1	m	68	S	GBM	4	r	y	n	0.5	na	na
Jawahar et al. (52)	2003	1	f	73	M	GBM	4	b	n	n	6	na	na
Salvati et al. (10)	2003	25	m15/f10	53(31-68)	S21M4	GBM18/AA7	4/3	r15/b10	y21/n4	y19/n6	8(0.5-18)	na	na
Kaku et al. (53)	2004	1	m	45	na	AA	3	r	y	y	Alive at 5	na	na
Saikali et al. (54)	2005	1	f	30	S	PA	1	r	n	y	36	na	na
Iza et al. (55)	2006	1	f	62	M	GBM	4	r	y	y	26	na	na
Ampil et al. (56)	2007	1	na	56	S	AA	3	b	y	n	2	na	na
Tsutsumi et al. (31)	2008	1	f	8	M	DA&GBM	2&4	r	n	y	5	na	na
Colavolpe et al. (57)	2008	1	m	44	S	GBM	4	r	n	y	18	MGMT, CD133	na
Vergani et al. (58)	2009	1	f	23	S	DA&O	2&2	r	n	y	alive at 84	loss of 19q	na
Salunke et al. (59)	2010	1	m	50	S	GBM	4	r	y	n	18	na	na
Hassaneen et al. (60)	2011	9	m7/f2	48(na)	S5M4	GBM	4	r9	y5/n4	y5/n4	12.9(na)	na	na
Sakai et al. (61)	2011	1	m	20	S	PA	1	b	n	n	alive at 48	syn-, nf-, TP53 -	na
di Russo et al. (62)	2013	18	m8/f10	66.5(37-78)	S15M3	GBM14/AA4	4/3	r18	y7/n11	y17/n1	10(4-29)	na	na
Terakawa et al. (63)	2013	5	m3/f2	32(23-35)	S	O3,DA1,O&DA1	2,2,2&2	r5	n5	n5	all alive 30(11-138)	IDH1 mutation	na
Kanoke et al. (28)	2013	1	m	30	M	PA&OA	1&2	r	y	y	alive at 14yr	BRAF amplification in 1, BRAF V600E mutation in 1, IDH1 R132H in 1	na
Garcia et al. (64)	2013	1	f	38	S	O	2	r	n	n	alive at 36	1p/19q loss, 10q and 7p loss, IDH1 mutation	na
Wan et al. (65)	2014	1	m	47	M	GBM	4	r	y	y	3	na	na
Yan et al. (25)	2015	5	m4/f1	56(38-70)	S4M1	GBM5	4	r5	y4/n1	y4/n1	7(4-30)	MGMT,1p19q mutations in 1	na
Sridharan et al. (40)	2015	1	m	49	S	DA	2	r	n	y	alive at 24	GG: IDH1 wildtype; LGA: PDGFRA, APC(E582A), CHEK2, ETV6, MLL2, SDHB, SF3B1	na
Ma et al. (41)	2015	1	m	20	S	GG&PA	1&1	r	n	n	alive at 48	na	na
de Eulate-Beramendi et al. (33)	2016	1	f	83	S	GBM	4	r	n	n	0.5	na	na
Inoue et al. (66)	2016	1	m	27	S	GBM	4	b	y	y	9	TP53, EGFR	na
Schroeder et al. (67)	2016	1	m	47	S	GBM	4	r	y	y	12	TP53 R175H, HDAC2, MARCKS, HDAC2A/2B deletion, MTSS1 loss, MET amplification, EGFRvIII mutation	na
Corrivetti et al. (68)	2016	1	m	41	S	DA	2	r	n	y	alive at 12	IDH1 mutation, with no 1p19q codeletion; IDH1 mutation, with 1p19q codeletion	na
Cabrera-Aldana et al. (34)	2017	1	m	40	S	GBM	4	b	n	n	1.5	na	na
Abou-el-ardat et al. (9)	2017	6	m5/f1	70 (56–74)	S6	GBM	4	na	na	na	na	PTEN, TP53, EGFR, and CDKN2A/B	TERTp in 5,PTEN in 2, EGFR in 1,CDKN2A in 1
Grosu et al. (69)	2017	1	m	30	S	OA	2	b	n	n	0	IDH1 nuclear positive	na
Picart et al. (24)	2018	2	m	61-83	S2	GBM	4	b2	n2	n2	1.5(1-2)	MGMT,EGFR;MGMT	na
Hayes et al. (70)	2018	4	m3/f1	29(21-44)	S3/M1	DA2,DA&AA1,O&DA2	2,2&3,2&2	r4	y2/n2	y2/n2	all alive 7.2yr(5.7yr-10yr)	IDH1,TP53,ARTX	IDH1 R132H in 2, TP53 in 1
Lahmi et al. (71)	2019	3	m3	63(58-65)	na	GBM	4	na	y3	y3	5(4-7)	na	na
Kohno et al. (37)	2020	1	m	78	S	Epithelioid GBM	4	r	y	y	Alive at 6	TERTp	na
Guerrini et al. (72)	2021	16	m11/f5	67.5(44-83)	na	GBM14/AA1/AO1	4/3/3	b5/r11	y11/n5	y11/n5	7(1-24)	IDH1 in 1, ATRX in 3, EGFR in 15, TP53 in 14	na
Enomoto et al. (73)	2021	1	f	4	S	GBM	4	b	y	y	19	IDH wildtype	na
Agopyan-Miu et al. (74)	2021	1	m	23	S	O	2	r	y	y	alive at 27	IDH1,PDGFR-A	TERT promoter, IDH1 R132H in 1; TERT promoter, IDH1 R132G in 1

(AA anaplastic astrocytoma, AO anaplastic oligodendroglioma, DA diffuse astrocytoma, GA gemistocytic astrocytoma, GBM Glioblastoma multiforme, GG Ganglioglioma, O oligodendroglioma, OA oligoastrocytoma, PA pilocytic astrocytoma, b biopsy, r resection, S synchronous, M metachronous, OS overall survival, y yes, n now, m male, f female, na not available, H high, L low, yr years) Annotation: The 2021 WHO Classification of CNS Tumors has cancelled the terminology of GBM and AA, and substitutes them with Glioblastoma, IDH-widetype, or Astrocytoma, IDH-mutant, grade 3 and 4. However, due to the referring cases in the table are mostly reported before the publication of the new classification, the terminologies of GBM and AA are continued to use.

## Prognostic Factors

Pathological grading is the most important prognostic factor for MCG. The median overall survival (OS) time for high-grade MCG is 8 months (25); while the median progression-free survival (PFS) time is 30 months for the low-grade (36). It has been confirmed that surgical resection of at least one lesion is an independent factor for good prognosis for high-grade MCG (25, 60, 62, 68, 75, 76), and other factors that may affect the prognosis include KPS score (76), younger age (75), receiving radiotherapy (75), and chemotherapy (25). For low-grade MCG, surgery, radiotherapy, or chemotherapy has been found to have no significant effect on the prognosis (36).

## Genetic Background

Abou-EL-Ardat et al. (9) conducted a detailed genetic analysis of the multiple lesions of multicentric GBM, and made the following important findings: First, the genetic abnormalities of the lesions involve RTK/PI3K, TP53, or RB pathway, as well as EGFR and CDKN2A/B, indicate that these pathway molecules play a key role in the development and evolution of GB. Second, the abnormalities of these pathway molecules are all at a relatively late position in the development of the disease and are not the original genetic events, and only the deletion of the PTEN and the mutation of the TERTp are very early genetic events shared among all lesions. This study shows that, compared with low-grade glioma, the gene mutations involved in the early stage of GB have a stronger tumorigenic ability, and it involves two key genes to activate the disease process which is in line with the “two-hit hypothesis”. Unfortunately, this study did not use whole-genome sequencing, so it is impossible to determine whether there were earlier genetic events before PTEN deletion and TERTp mutation.

A study on the genetic background of low-grade MCG found that IDH mutations are very early events of genetic abnormalities in 3 clinical cases (two of them are the first events, and the other is the second event following the point mutation of TP53 in the germ cell line) (70). The results of this study show: 1. The use of genetic analysis in MCG can effectively screen the source gene mutations. 2. Some low-grade MCG share the same source mutations as solitary glioma. 3. The possibility does exist of other earlier genetic events before IDH1 mutations. The question that this study did not solve is, for low-grade gliomas that are negative for IDH1/IDH2 mutations, what is the source gene event? In another study, using immunohistochemistry, in 14 cases of MCG (including 4 cases of multicentric LGG), no IDH1-R132H mutation was detected (77), suggesting that some MCG could have other pathogenic mutations of origin.

When making traceability study of the genetic mutations in glioma with MCG, an unavoidable question is, whether MCG has universal significance or just represent a small special subtype of glioma. Considering clinical data, MCG is similar to solitary glioma in terms of age of onset, sex ratio, tumor location and pathological type. Current genetic studies have not found unique mutations in MCG comparing with solitary ones (9). Therefore, it is supported by many authors that the occurrence of MCG is more due to changes in cell behavior rather than specific gene mutations. On the other hand, considering MCG as a group, some genetic features were found in them that are different from solitary gliomas, such as different promoter methylation and gene expression patterns (78, 79). However, the different expressed genes found by now are not specifically mutated or expressed in MCG, and the difference of their expression pattern is possibly because MCG represents a special stage in the progression of glioma. Moreover, for metachronous MCG, the initial lesion presents as single and has the same manifestations with solitary gliomas. Therefore, although MCG have their particularities, MCG may still share the same source of genetic aberrance with solitary gliomas, and it still have universal significance to make traceability study of the gene abnormalities in MCG.

The 2021 WHO Classification of CNS Tumors has included pediatric-type glioma into the scheme of gliomas. However, due to most of the clinical and genetical studies of MCG reported are focusing on adult patients, in the following, we will stress on the discussion of adult-type diffuse glioma. The terminology of glioma in the following paragraphs, is mainly referred to this type of glioma.

## Early Events of Genetic Aberrance in Glioma

A mutation in IDH1/2 is considered as an early event in glioma-genesis. Point mutations in the IDH1/2 gene can cause changes in the activity of NADP^+^-dependent isocitrate dehydrogenase and cause the accumulation of abnormal metabolites, leading to the occurrence of Astrocytoma, IDH-mutant, grade 2-4 (80). IDH mutations are often accompanied by TP53 mutations, suggesting that there may be some interactions between them. IDH1-R132H is the most common type of IDH1 mutation, accounting for 90% of all IDH1 mutations, which is related to the concentration of metabolites produced by this site mutation that is just conducive to tumor cell formation (22).

TERTp mutation is an early event in the pathogenesis of GB (81). Normal telomere length is considered to be an important limit for controlling the number of cell replications. Point mutations in the TERT promoter region can increase the transcription of TERT enzyme, extend the telomere length of cells, and enable tumor cells to gain unlimited replication. In addition to the TERTp mutation, Alpha thalassemia/mental retardation syndrome X-linked (ATRX) or death-domain associated protein (DAXX) have been shown to underlie a telomere maintenance mechanism not involving telomerase. It has been reported that 83% of primary GBs are associated with increased TERT activity (82). In addition, cancers generated in tissues with relatively low rates of self-renewal, including melanomas, liposarcomas, hepatocellular Carcinomas, urothelial carcinomas, squamous cell carcinomas of the tongue, medulloblastomas, are also accompanied by increased TERT activity (83).

PTEN loss may also be at the source of glioma. In some animal experiments, knocking down PTEN in neural precursor cells combined with changes in the expression of other key genes can induce gliomas (84). A study has reported PTEN loss as an early event in whole exome and transcriptome multi-focal sequencing of a case of diffuse intrinsic pontine glioma (85).

It should be pointed out that there are still many gliomas that have not clarified with clear early genetic events. Moreover, the occurrence of tumors may be an individualized phenomenon. For each tumor, its origin has some common features, but there will be some unique mutations. It will help to find these early mutations by molecular genetic evaluation and by analyzing evolutionary relationship between different foci in individual case. Multicentric gliomas will undoubtedly be of great help to this kind of researches.

## Gene Mutation, Cell Stemness and Genesis of Glioma

Promoting the expression of some oncogenes or silencing some tumor suppressors by transgene technologies could lead to the formation of astrocytomas *in vivo* in animal models. The oncogenes that have successfully induced high-grade astrocytoma include Ras, Akt, EGFR, PDGFR, and often in combination with mutations in tumor suppressors such as Ink4A or Arf (86). The tumor suppressor genes used in animal models include NF1, TP53, and PTEN (84, 87, 88). Mutations of the genes mentioned above are frequently found in astrocytomas and glioblastomas. The dysfunction of the genes could disrupt cell cycle and apopsis regulation (INK4A, TP53, Arf), and growth factor receptor signaling (PTEN) (89). Patients with mutation of NF1 in germline are predisposed to suffer from astrocytoma, neurofibroma, and GB (90). The fact that changing the expression of oncogenes or tumor suppressor genes could introduce the formation of glioma in normal brains in animal models, suggests that if the tumorigenic ability of the mutations reaches to a certain level, it could cause glioma genesis in normal brain.

**Figure 2:**
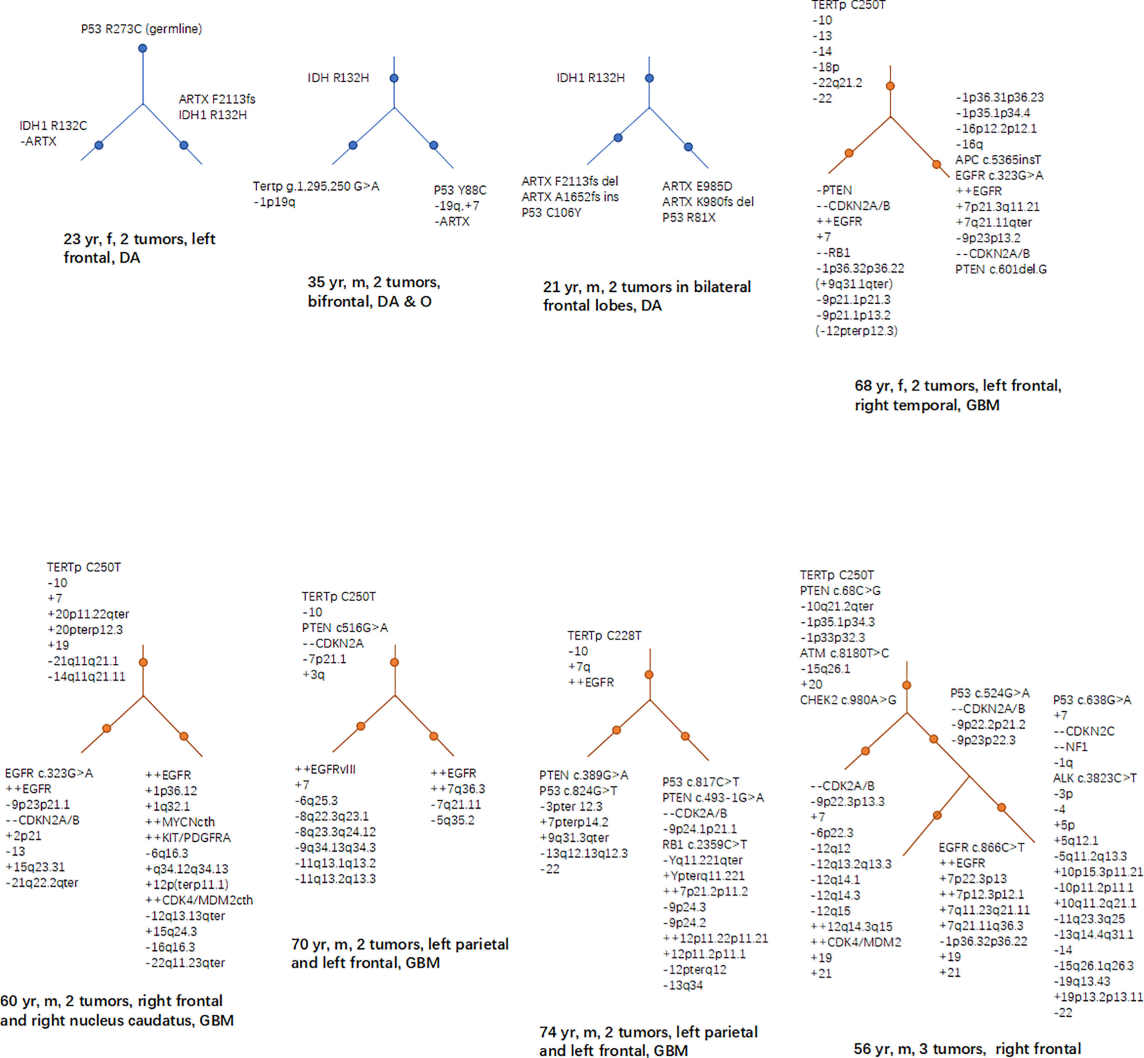
Hierarchical structures of the mutations revealed by studies based on MCG cases. In low grade glioma (including diffuse astrocytoma and oligodendroglioma), mutations of IDH, TP53 and ARTX frequently lies in the higher levels of the hierarchy; while in GB, TERTp, PTEN loss, EGFR, and CDKN2A are more frequently seen in the higher levels of the mutational hierarchy.

The mutations of some genes that manipulated by human could lead to glioma genesis, doesn’t mean that they are also “start-up” mutations in the natural status of glioma. What needs to be further answered is, in the natural pathological conditions, which mutations play a key role at early stages in causing normal cells to transform into tumor cells. A study has found that in the tumor-free SVZ region of GB patients, some neural precursor cells carried shared driver mutations with their matching tumor, including TERTp mutation and single nucleotide variants of TP53, EGFR, PTEN, and/or RB1 (91). Studies based on clinical cases of MCGs have found that certain mutations, such as IDH1/2, TERTp, PTEN, and TP53, EGFR, CDKN2A in a less proportion, are more often to occur at early stages of tumor evolution (**Figure 2**) (9, 70, 71). We suppose that source mutations in normal cells could lead to the mutation and dysfunction of downstream oncogenes or tumor suppressors through accumulations of gene mutations, and the course is accompanied with the process of glioma genesis and development.

Another phenomenon revealed by animal model experiments is, compared with mature cells, stem and precursor cells are easier to be transformed into tumor cells. High-grade gliomas can be induced by silencing the expression of TP53, Nf1 and PTEN in neural stem/progenitors in the SVZ or SGZ of mice brains (84, 87, 88). When silencing the expression of these genes in mature neural cells in non-neurogenic brain regions (such as cortex and striatum), different studies have drawn different conclusions: some studies have found it cannot form gliomas (84); while other studies have got the opposite results (88). This may be explained by the different techniques and experimental conditions in the experiments. However, these results at least suggest that, it is more difficult to induce mature neural cells into glioma cells compared to neural stem cells and precursor cells. We speculate that whether a normal cell can be transformed into a tumor cell is related to the stemness of the target cells and the tumorigenic ability of the mutations. Compared with mature cells, stem and precursor cells are more likely to be affected by tumorigenic mutations and transforms into tumor cells. However, when the tumorigenic ability of the mutations reaches a certain level that is strong enough, even mature cells can be induced into tumor cells as well.

## Migration of Neural Stem Cells and Its Roles in the Genesis of MCG

The neural stem cell pool of adult human is mainly located in the SVZ (18, 92–95). In human periventricular heterotopia, an X-linked dominant genetic disease, the normal migration and differentiation of neural stem cells in this area is affected and thus forms many tumor nodules around the ventricles, combined with hypogenesis and smaller sizes of the corpus callosum, brain stem and cerebellum (19, 96–98). This proves from the side that the SVZ is the source of stem cell migration and differentiation *in vivo*. Microstructure of SVZ consists of several layers. The ependyma forms the first layer, below which is the gap area lacking cells. This area in rodents is rich of precursor cells and the activation, replication, and division of these cells mainly forms neuroblasts of the rostral migratory stream(RMS) (99–104). In human embryos and infants, similar activated cells exist in this area, but in human adults, this area forms an oligocellular zone (105). Below the gap is a dense band of cells rich in astrocytes, which are considered to be the main source of adult neural stem cells (106). Some astrocytes of this layer will extend their synapses to the ependymal cell layer and receive molecular signals in the cerebrospinal fluid (CSF) (107–109). In addition, there are some axons and synapses from distant neurons distributed in this layer and contacting with the astrocytes (92). Therefore, it is speculated that the activation of neural stem cells may be regulated by the neuro-psychological-endocrine network. Below the astrocyte layer is a transition zone lacking cells, and then to the outside is the brain parenchyma (92, 108).

**Figure 3:**
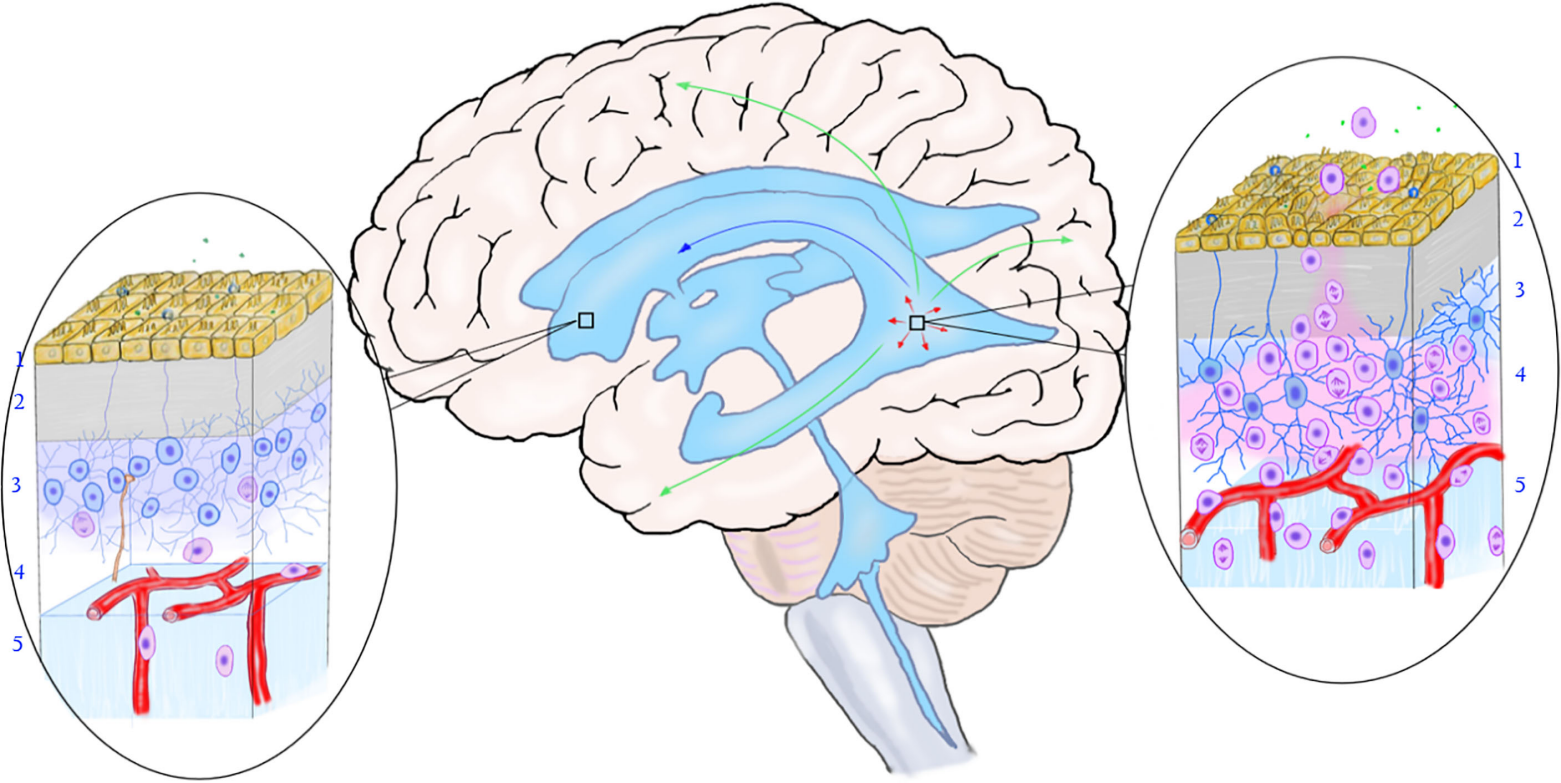
The relationship between the mutation of neural stem cells in the subependymal zone and the occurrence of multicentric glioma. The rectangular box on the left shows the normal subependymal area that is not affected by the tumor, and the oval area on the left shows the normal microstructure of the subependymal area. From the ventricle to the brain parenchyma, there are ependymal layer (layer 1), gap zone (layer 2), astrocyte zone (layer 3), loose layer (layer 4), and brain parenchyma (layer 5). It can be seen that neural stem cells differentiate into less primary progenitor cells, and the local cell hierarchy is regular. The rectangular frame on the right shows the location of the mutant neural stem cells, and the oval area on the right shows the microstructure of the area. As shown in the figure, early gene mutations occurred in neural stem cells in this area, leading to active cell proliferation and differentiation into progenitor cells. The primary progenitor cells migrated along the white matter fibers and blood vessels into the brain parenchyma, replicated, differentiated, and accumulated mutations to form tumors. The arrow shows the migration pathway of the mutant progenitor cells: the green arrow shows the migration into the brain parenchyma, the red arrow shows the local migration along the subependymal zone, and the blue arrow shows the migration along the cerebrospinal fluid (to be verified). (Yellow cells in layer 1, ependymal cells; blue cells in layer 2, neural stem cells; pink cells in layer 3, progenitor cells; brown-yellow cords in layer 3 and layer 4, axons and synapses of distant neurons; green dots, chemical transmitters in cerebrospinal fluid).

Based on the reported distribution of MCG lesions, it is speculated that the following migration pathways may exist in the SVZ area: 1. Mutations occur in some neural stem cells or precursor cells in SVZ, making them proliferate and form nest there; 2. Mutated precursor cells of the nest in SVZ migrate and differentiate along white matter fibers and blood vessels (110, 111) in one or more directions, forming solitary or multiple gliomas in the brain parenchyma; 3. Neural stem cells in the subependymal zone and the migration site of the cortex exist mosaic-like correspondence (112), which may explain why MCG in different parts of the brain have various migration patterns (113); 4. The nest grows and spreads along the subependymal area, gradually involving more subependymal areas. 5. The mutated neural stem cells in the nest migrate into the CSF, colonize with the drift of the CSF to other areas of the ventricle wall, and form a new nest in the local SVZ (**Figure 3**).

**Figure 4:**
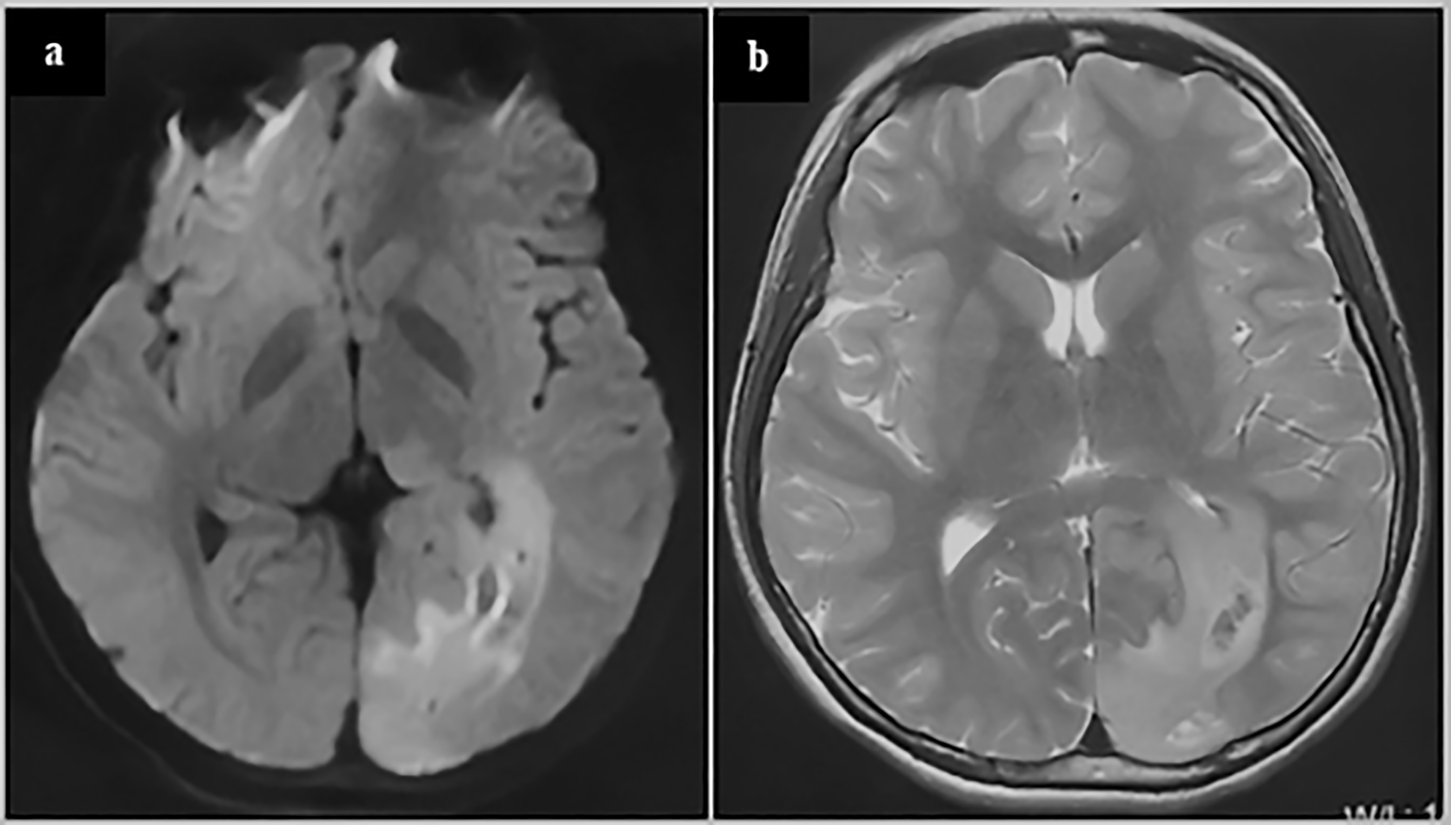
glioblastoma of a 13-year-old adolescent. On the MRI FLAIR **(A)** and T2 **(B)**, abnormal signals can be seen spreading from around the occipital horn of the ventricle to the occipital cortex. These abnormal signals may reflect the process of the migration, replication and differentiation of the mutant precursor cells from the subependymal zone to the mature brain lobe. The patient underwent a subtotal tumor resection. Postoperative pathology revealed GBM. Immunohistochemistry showed Ki67 20%+, IDH1-, TP53 weakly+, Olig2-, H3K27M-, H3K27me3+, and ATRX partially missing. The patient was given local radiation and temozolomide based chemotherapy after surgery and is currently under follow-up (3 months after surgery).

The above speculated MCG migration pathway is supported by some experimental and clinical evidence. A study detected early genetic changes homologous to the tumor in neural stem cells in the SVZ that were not invaded by tumors, suggesting that there are abnormal neural stem cell nests in the SVZ that is not yet invaded by tumors (91). By analyzing the single-cell landscape of shared and tumor-private mutations in the tumor and early nests in SVZ of patient, they found that the clonal evolution of cells harboring driver mutations in the direction from SVZ to GB instead of the opposite direction. Using a mouse model of TP53, PTEN and EGFR mutations in NSCs from the SVZ through genome editing, which were recurrent driver mutations found in the tumor-free SVZ tissues from the patients with GB, the early abnormal neural stem cell nests were induced in the subependymal zone, and glioma cells could be detected in multiple areas in the mouse brain. In addition, to examine the possibility of whether cells harboring shared driver mutations spread to the SVZ at a very early stage of disease, the authors introduced TP53, PTEN and EGFR mutations into the cortex. After the stereotactic injection, neither spread of cells to the SVZ nor statistically significant proliferation of cells was noted, compared to the mouse model carrying driver mutations in SVZ. The findings suggest that in pathological conditions of glioma patients, the nests of abnormal neural stem cells are formed at SVZ and can migrate to multiple areas in the brain parenchyma to form gliomas, instead of that the nests formed at cortex and migrate to the SVZ. In clinical cases, changes in MRI T2 or FLAIR may indicate the migration trajectories of mutant cells. In some cases, there are obvious abnormal signals from the ventricle wall to the cerebral cortex on MRI T2 or FLAIR, which may reflect the migration path of the mutant cells from SVZ to the cerebral cortex (**Figure 4**). Although there is no direct evidence that mutated neural stem cells can migrate with the CSF, neural stem cells can also be isolated and cultured in the CSF of the human embryos and premature infants (114–116). This shows that neural stem cells can exist in the CSF in the early stages of life. In addition, in the CSF of patients with GB, a complete genome homologous to the tumor was detected and shared the same early gene mutations (including IDH and TERTp) with the tumor (117). Therefore, we speculated that in a tumor state, some mutated neural stem cells may regain abilities in early stages of life, making them migrate into, survive in and may spread with the CSF. This can explain some bilateral or supra-subtentorial MCG.

## Discussion (Our Perspective)

In the past, the histopathological grade of the tumor was usually used to reflect the degree of malignancy of the tumor. However, for some gliomas with the same pathological grade, the tumor characteristics and patient survival period can be very different. For example, multicentric GBs have a worse prognosis than solitary ones. What’s more, the IDH1/2 mutant type has a better prognosis than the IDH1/2 wild type in glioma with the same pathological grades. These evidences show that in addition to the histopathological grade, there are other factors involved in determining the degree of malignancy of glioma.

Some clinical phenomena of glioma suggest that the malignant degree of glioma is related to the stemness of the precursor cells of origin. The phenomenon of gliomas of WHO grade I and of some grade II may be cured after surgery, indicates that the source mutation may occur in late progenitor cells or even mature glial cells in these gliomas. On the other hand, the existence of multicentric low-grade gliomas indicates that even in low-grade gliomas, the source of mutation can appear in neural stem cells or early progenitor cells. This may explain why diffuse astrocytomas are difficult to be cured, and will relapse and progress. The aggressiveness and relapsing tendency of high-grade gliomas and the fact that they are more likely to have multicentric lesions than low-grade gliomas suggest that the source of high-grade gliomas may originate from neural stem cells or early precursor cells. Theoretically, stem cells survive for a long time and repeatedly replicate and divide, which can lead to accumulation of genetic mutations, and therefore are more prone to accumulate tumorigenic mutations. With growing of age or under some conditions of chronic inflammations, the replication of stem cells increases, which can explain why malignant tumors is more likely to occur in the elderly or in patients with some chronic inflammatory conditions (such as chronic hepatitis and gastritis). In the nervous system, with age increases, the number and replication activity of neural stem cells also decline, which may explain why the incidence of glioma has a curve with age of first rises and then declines. The incidence of GB peaks at the age from 40 to 60, and decreases in the elderly.

In addition to cell stemness, some clinical and laboratory evidence also suggests that the malignancy of glioma is related to the tumorigenic ability of early mutant genes. IDH1/2 mutation can cause some low-grade gliomas and secondary GBs, which indicates that certain low-tumorigenic mutations can cause gliomas with a relative slower evolution tendency. The phenomenon of some low-grade gliomas will gradually progress to high-grade gliomas may reflect the gradual accumulation of genetic mutations during tumor progression. When key genes closely related to the cell cycle and signal pathways are involved, the malignancy of tumors will increase significantly. The mutations of the primary GB may occur in key genes related to the cell cycle or signaling pathways at an early stage, so the tumor shows high malignancy even at the early stage of the tumor. What’s more, according to the results of some studies, at least two key gene are involved in early stage of GB [e.g. TERTp mutation and PTEN deletion (35)], which is consistent with the hypothesis of “twice hits” in the mechanism of malignant tumorigenesis.

**Figure 5:**
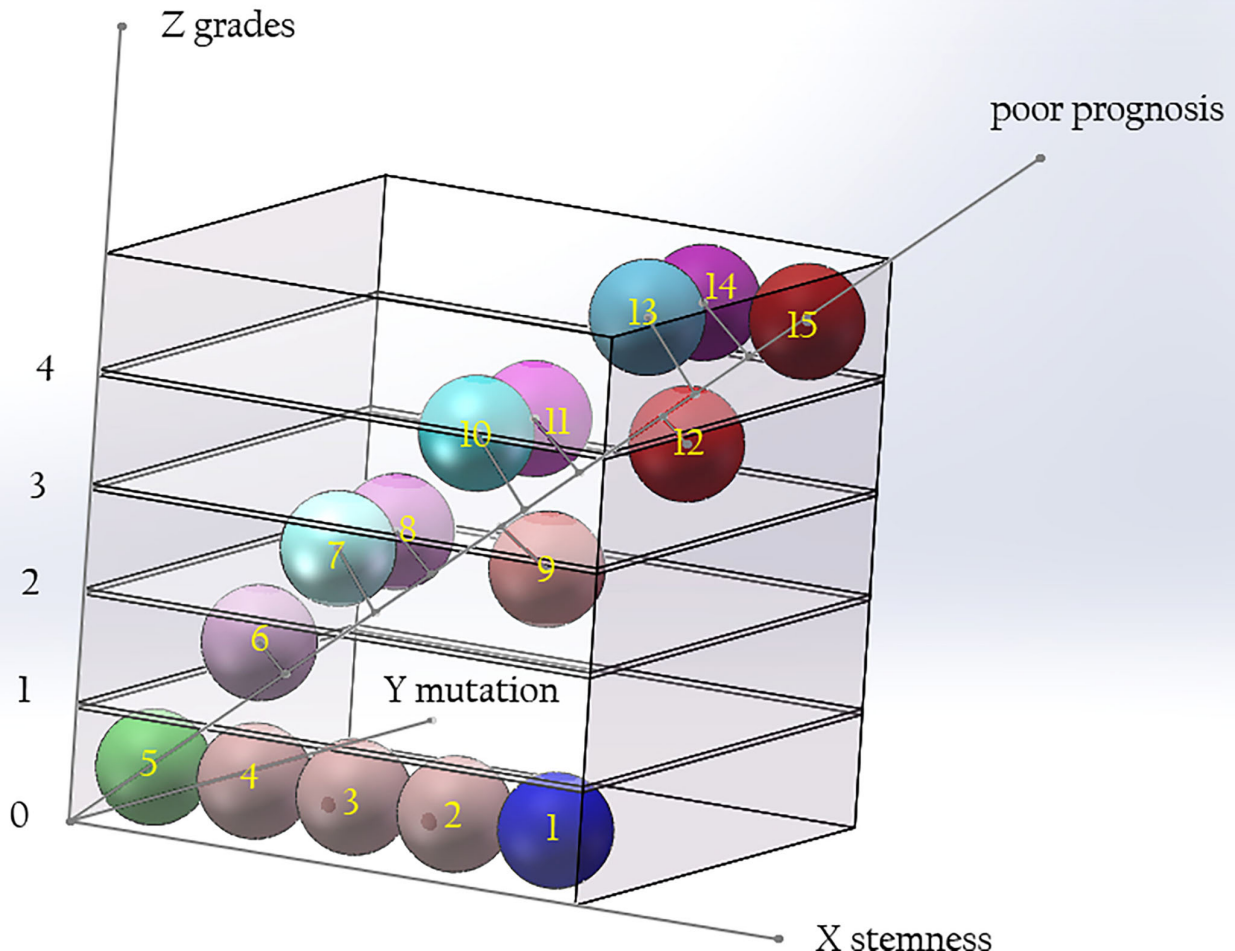
The relationship among the grade of gliomas, the tumorigenicity of mutations, and the stemness of originate cells. The malignancy of glioma is determined by the tumorigenic ability of gene mutation and the stemness of originate cells. With the improvement of the tumorigenicity of the mutant genes and the increase of the stemness of the originated cells, the grade of the tumor increases. Compared with solitary gliomas of the same grade, MCG have similar tumorigenicity of mutant genes, but the stemness of the originated cells is increased. The diagonal of the cube signifies poor prognosis. Along the direction far from 0, the prognosis of patients will be worse. (X axis: stemness of originated cells; Y axis: tumor grade; Z axis: tumorigenicity of mutant genes. 1, neural stem cell; 2-4, precursor cells; 5, mature neural cells; 6, glioma of WHO grade I; 7, diffuse astrocytoma/oligodendroglioma, IDH mutant, grade 2; 8, diffuse astrocytoma, IDH wide type, grade 2; 9, MCG, grade 2; 10, diffuse astrocytoma/oligodendroglioma, IDH mutant, grade 3; 11, diffuse astrocytoma, IDH wide type, grade 3; 12, MCG, grade 3; 13, diffuse astrocytoma, IDH mutant, grade 4; 14, glioblastoma, IDH wide type, grade 4; 15, MCG, grade 4.).

We believe that the malignant degree of a tumor is determined by the histopathological grade, the stemness of the cells of origin, and the tumorigenic ability of the early mutant genes. If the above three factors are represented by the X, Y, and Z axes, a three-dimensional coordinate system can be drawn. The diagonal line passing through the Origin represents the degree of malignancy of the tumor, which is negatively correlated with the median survival time of patients (**Figure 5**). With the improvement of the tumorigenic ability of the mutant gene and the increase of the stemness of the tumor-originating cells, the degree of malignancy of the tumor gradually increases, and the median survival time of patients gradually shortens. Different types of gliomas can be marked at different positions in the coordinate system. If a vertical line is drawn from their position to the diagonal line passing through the origin, the projection position on the diagonal line can reflect the malignant degree of the tumor. Highly differentiated gliomas, such as WHO grade I gliomas, may originate from precursor cells with a low stemness, and their early mutant genes have low tumorigenicity, so their positions in the coordinate system are near from the origin, and the malignancy of the tumor is low. Astrocytoma, IDH-mutant, grade 4 (also called as secondary GB in old classification systems) has IDH1/2 mutations with low tumorigenicity. Compared with GB, its early mutant genes have lower tumorigenicity. Therefore, it is marked on the lower side of primary GB in the stemness axis of the coordinate system. Although the pathological grades of secondary GB are the same as primary ones, their projection on the diagonal is nearer to the origin, reflecting a lower degree of malignancy. Compared with solitary gliomas of the same grade, MCG may have similar tumorigenicity of the mutant genes, but the stemness of the originate cells is higher. Therefore, their projection position in the diagonal line is farther form the origin compared with solitary gliomas. This explains why the prognosis of MCG is worse than that of solitary gliomas of the same grade.

## Author Contributions

YY and QM contributed to conception. YY and WD organized the database. YY wrote the first draft of the manuscript. YY, WD, and QM wrote sections of the manuscript. All authors contributed to manuscript revision, read, and approved the submitted version.

## Funding

This work was supported by Natural Science Foundation of China (NO. 81101908) and Medical Guidance Science and Technology Support Project of Shanghai Municipal Science and Technology Commission (NO.19411968400).

## Conflict of Interest

The authors declare that the research was conducted in the absence of any commercial or financial relationships that could be construed as a potential conflict of interest.

## Publisher’s Note

All claims expressed in this article are solely those of the authors and do not necessarily represent those of their affiliated organizations, or those of the publisher, the editors and the reviewers. Any product that may be evaluated in this article, or claim that may be made by its manufacturer, is not guaranteed or endorsed by the publisher.
